# Supplementary material for: Examining the effects of time of day and sleep on generalization
Source: PLoS One. 2021 Aug 2;16(8):e0255423. doi: 10.1371/journal.pone.0255423 (PMC8328323; doi:10.1371/journal.pone.0255423)
Supplement: S1 Table — (PDF) [file pone.0255423.s001.pdf]

S1 Table. Training performance.

		Number of blocks to criterion		Feature accuracy last block		Class accuracy last block	Participants who did not meet training criterion
		**Initial**	**Bridge**	**Initial**	**Bridge**	**Initial**	**Initial**
**Exp. 1**	Morning	4.15±0.36	2.46±0.27	0.73±0.02	0.95 ±0.01	0.94±0.03	1
	Evening	4.94±0.50	2.59±0.32	0.73±0.02	0.94±0.01	0.96±0.01	2
	Group Diff. *p*	.24	.77	.96	.56	.63	
**Exp. 2**	Morning	4.36±0.48	1 (fixed)	0.71±0.02	0.69±0.06	0.98±0.01	1
	Evening	3.77±0.46	6 (fixed)	0.73±0.03	0.98 ±0.01	0.98±0.01	2
	Group Diff. *p*	.38		.59	.000005***	.61	
**Exp. 3**	Morning	4.07±0.32	2.71±0.27	0.73±0.02	0.94±0.01	0.96±0.01	1
	Evening	4.38±0.27	2.54±0.31	0.76±0.01	0.96±0.01	0.97±0.01	0
	Group Diff. *p*	.46	.67	.38	.35	.56	
**Exp. 4**	Nap	4.45±0.44	2.10±0.14	0.76±0.02	0.97±0.01	0.98±0.01	2
	No Nap	3.94±0.39	2.52±0.23	0.68±0.04	0.95±0.01	0.96±0.02	3
	Group Diff. *p*	.40	.11	.07	.30	.65	
**Exp. 5**	Morning	4.36±0.41	2.28±0.11	0.77±0.02	0.95±0.01	0.98±0.01	2
	Evening	4.39±0.45	1.94±0.17	0.76±0.02	0.97±0.01	0.98±0.01	0
	Group Diff. *p*	.96	.09	.72	.35	.75	
**Exp. 6**	Morning	4.12±0.33	2.31±0.16	0.74±0.01	0.96 ± 0.01	0.97±0.01	1
	Evening	5.57±0.47	2.52±0.23	0.76±0.02	0.95 ± 0.01	0.96±0.02	1
	Group Diff. *p*	.01*	.43	.17	.50	.77	
**Exp. 7**	Morning	4.34±0.22	2.17±0.11	0.68±0.02	0.95±0.01	0.96±0.01	9
	Evening	3.74±0.21	2.21±0.11	0.70±0.02	0.97±0.01	0.94±0.02	5
	Group Diff. *p*	.05*	.80	.67	.07	.31	

Measures of performance during the main training phase (Initial) and the following bridge training phase (Bridge) are shown for each experimental group. Number of blocks to criterion = The number of blocks it took to reach the training criterion (Initial training criterion was 66% for feature accuracy and 90% class accuracy), or the number of blocks after 1-hour had passed; Bridge training criterion was 90% feature accuracy. Feature accuracy last block = Proportion of features correct in the last block of the initial and bridge training phase. Class accuracy last block = Proportion of trials correct on class naming in the last block of the initial training phase. Participants who did not

meet training criterion = The number of participants that did not reach the training criteria in the initial training phase after 1-hour had passed. Descriptive statistics (mean ± one SEM) and group differences (*p*-values from t-test) are shown. Note that the number of trials per block in the initial training phase was lower in Exp. 5 and Exp. 6. ***$p < .001$, *$p < .05$.
