# Supplementary material for: Examining the effects of time of day and sleep on generalization
Source: PLoS One. 2021 Aug 2;16(8):e0255423. doi: 10.1371/journal.pone.0255423 (PMC8328323; doi:10.1371/journal.pone.0255423)
Supplement: S2 Table — (PDF) [file pone.0255423.s002.pdf]

S2 Table. Survey measures.

		MEQ Score	MEQ Chronotype Proportions (Morning /Neutral/ Evening)	SSS	H Since Wake	Sleep Qual. Last Night	H Asleep Last Night	Epworth
**Exp. 1**	Morning			2.85±0.22	2.01±0.21	2.00±0.20	6.92±0.19	6.92±1.12
	Evening			2.35±0.15	11.77±0.40	2.06±0.16	8.35±0.34	5.53±0.72
	Group Diff. *p*			.06		.82	.002**	.29
**Exp. 2**	Morning			2.36±0.23	1.76±0.16	1.64±0.20	7.66±0.32	6.07±0.66
	Evening			2.61±0.27	12.22±0.32	2.15±0.25	7.56±0.38	7.85±1.07
	Group Diff. *p*			.46		.12	.83	.16
**Exp. 3**	Morning	47.93±2.65	21% / 50% /29%	2.14±0.18	1.93±0.16	2.07±0.16	7.35±0.32	5.50±0.87
	Evening	50.46±2.65	15% / 70% /15%	3.23±0.43	13.01±0.86	1.92±0.14	7.33±0.36	5.69±0.93
	Group Diff. *p*	.51		.02*		.50	.96	.88
**Exp. 4**	Nap	46.90±2.46	10% / 60% / 30%	2.50±0.18	8.02±0.26	1.85±0.13	7.40±0.30	7.20±0.63
	No Nap	48.65±2.47	24% / 47% / 29%	2.35±0.17	7.81±0.43	2.12±0.17	7.60±0.27	6.47±0.51
	Group Diff. *p*	.62		.58	.66	.21	.62	.39
**Exp. 5**	Morning	49.08±1.55	8% / 72% /20%	2.00±0.12	1.94±0.16	1.80±0.16	7.09±0.15	5.92±0.55
	Evening	51.56±2.11	17%/ 67% /17%	2.44±0.25	13.10±0.23	2.06±0.19	7.77±0.25	6.06±0.86
	Group Diff. *p*	.34		.08		.31	.02*	.89
**Exp. 6**	Morning	49.81±1.68	15% / 69% / 15%	2.08±0.17	2.02±0.18	1.88±0.14	7.51±0.20	6.62±0.65
	Evening	50.55±2.30	14% / 59% / 27%	2.68±0.20	13.43±0.27	1.73±0.15	7.82±0.15	4.95±0.62
	Group Diff. *p*	.79		.03*		.45	.23	.07
**Exp. 7**	Morning	48.29±1.23	10% / 63% / 27%	2.22±0.12	1.77±0.09	1.73±0.09	7.41±0.12	6.46±0.60
	Evening	45.81±1.22	3% / 76% / 22%	2.38±0.17	12.25±0.21	1.76±0.10	7.86±0.15	6.32±0.55
	Group Diff. *p*	.16		.45		.85	.03*	.86

Group descriptive statistics (mean ± one SEM) and Morning and Evening group comparisons (t-tests) for each experiment are shown. For two session experiments (Experiment 1, Experiment 2, Experiment 3) only Session 1 survey responses are presented. MEQ Score = score on the Morningness-Eveningness Questionnaire, with higher values reflecting a circadian preference towards the morning, MEQ Chronotype Proportions = assigned chronotype

using the standard Morningness-Eveningness Questionnaire scoring, percentages are rounded to the nearest whole number, SSS = participant response on the Stanford Sleepiness Scale, with higher values indicating more sleepiness, H Since Wake = number of hours since wake time in the morning (calculated from participant reported wake time), Sleep Qual. Last Night = participant reported quality of sleep the night prior to the experiment (1 = Excellent, 2 = Good, 3 = Fair, 4 = Poor.), H Asleep Last Night = participant reported hours spent asleep the night prior to the experiment, Epworth = score on the Epworth Sleepiness Scale, with higher values indicating more daytime sleepiness.**$p < .01$, *$p < .05$. In Experiment 3, the Evening group in this sample was sleepier than the Morning group. This difference did not relate to generalization in regression models (*p*'s > .16).
