# Supplementary material for: Examining the effects of time of day and sleep on generalization
Source: PLoS One. 2021 Aug 2;16(8):e0255423. doi: 10.1371/journal.pone.0255423 (PMC8328323; doi:10.1371/journal.pone.0255423)
Supplement: S2 Fig — (PDF) [file pone.0255423.s004.pdf]

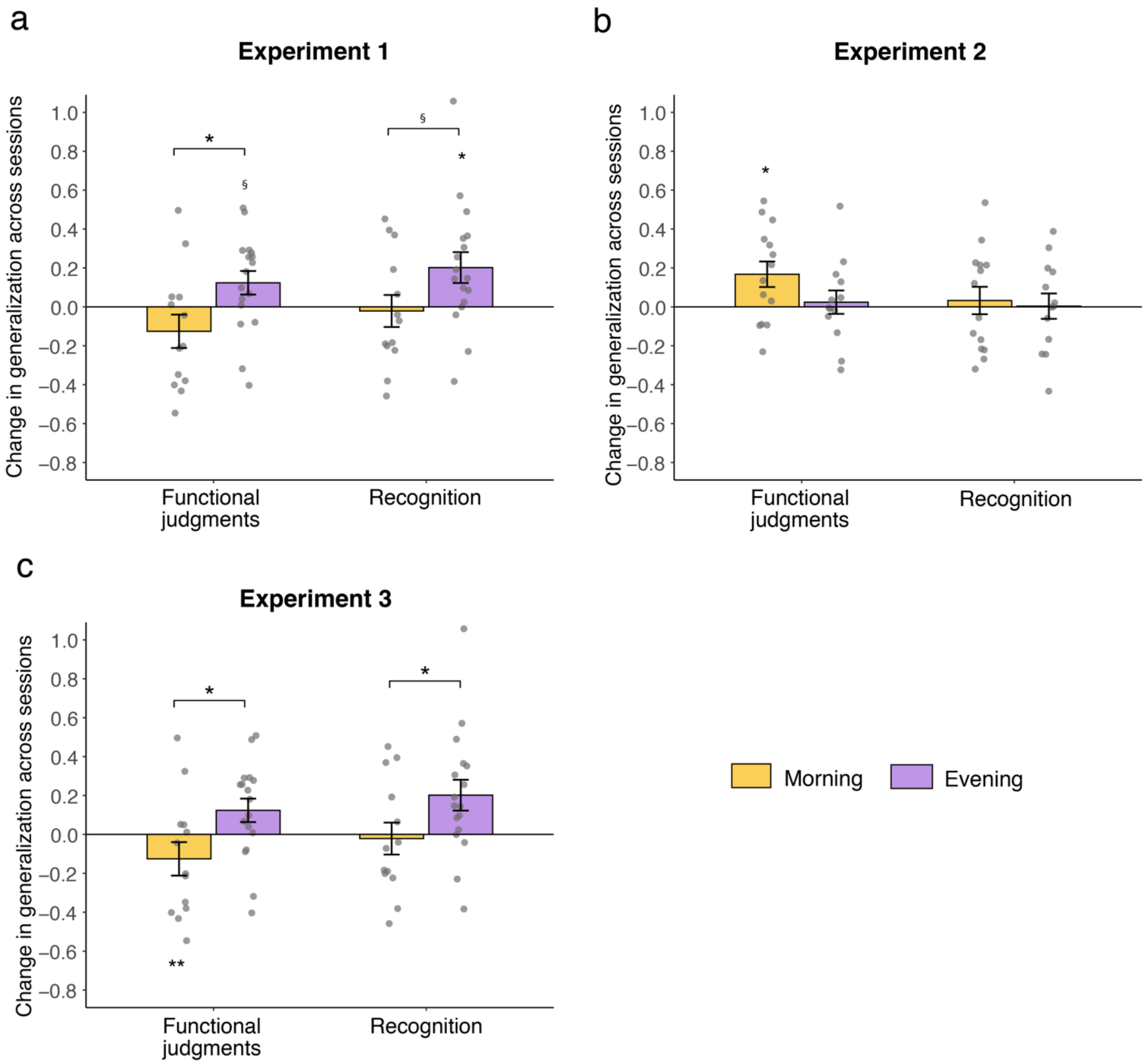

S2 Fig. Change in generalization across sessions (Test 2 Generalization – Test 1 Generalization). (a) Experiment 1, (b) Experiment 2, and (c) Experiment 3. For Experiments 1 & 2 (12h delay), the Morning group was tested in the morning in Test 1 and evening in Test 2, and the Evening group was tested in the evening in Test 1 and morning in Test 2. For Experiment 3 (24 hour delay), the Morning group was tested in the morning on both Test 1 and Test 2, and the Evening group was tested in the evening on both Test 1 and Test 2. *$p < .05$, §$p < .1$
