# Supplementary material for: Examining the effects of time of day and sleep on generalization
Source: PLoS One. 2021 Aug 2;16(8):e0255423. doi: 10.1371/journal.pone.0255423 (PMC8328323; doi:10.1371/journal.pone.0255423)
Supplement: S3 Fig — (PDF) [file pone.0255423.s005.pdf]

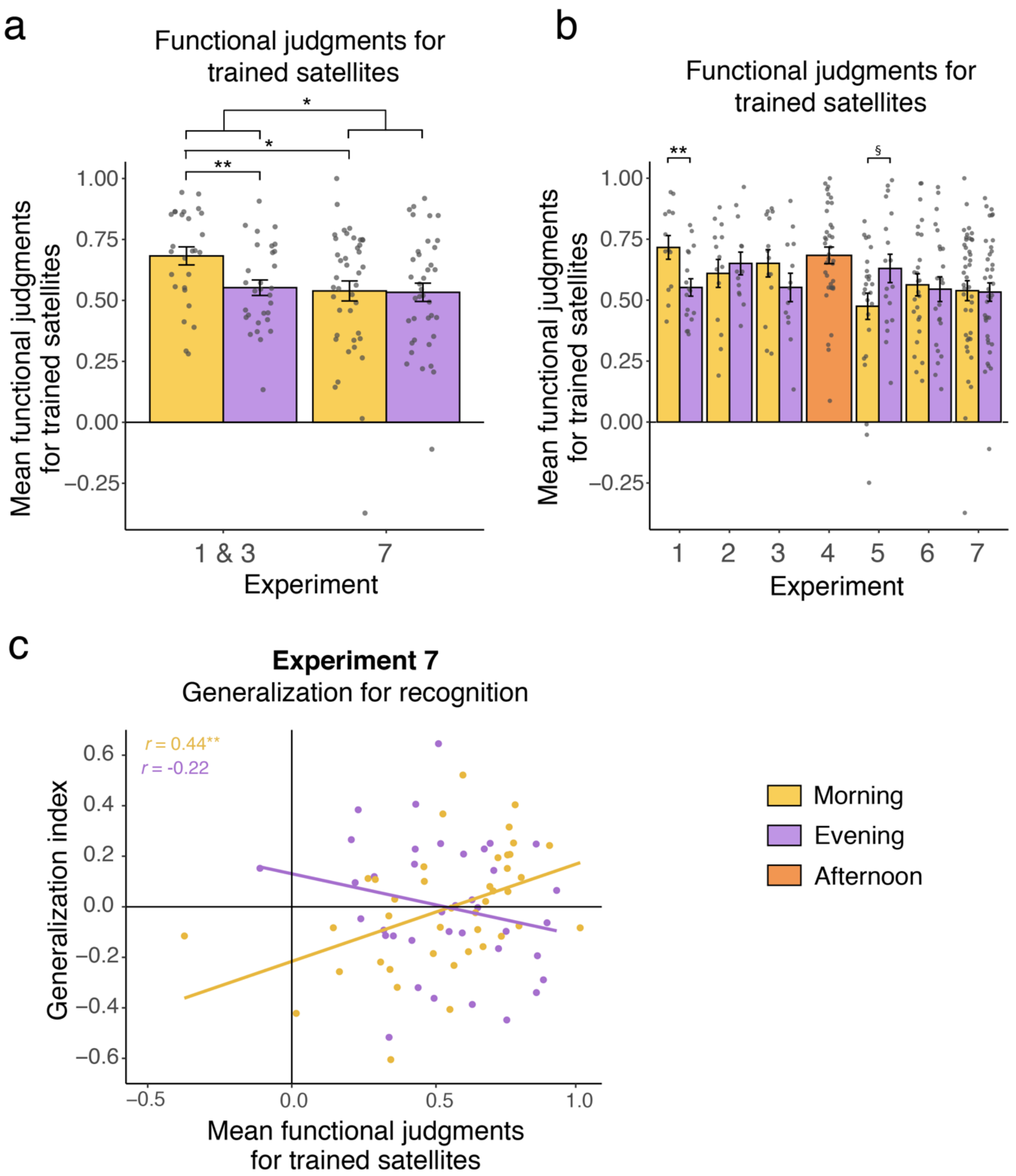

S3 Fig. Functional judgments for trained satellites. (a) Functional slider judgments for trained satellites in Experiment 1&3 (pooled) and Experiment 7. (b) Functional slider judgments for trained satellites across all experiments. The reliable Morning vs. Evening group differences within experiments are marked. (c) Interaction between time of day and functional judgments for trained satellites and generalization for recognition in Experiment 7. Post-hoc Pearson's correlations were computed separately for the Morning and Evening group and are depicted in the top left quadrant. *$p < .05$, ** < .01 §$p < .1$
