# Supplementary material for: Examining the effects of time of day and sleep on generalization
Source: PLoS One. 2021 Aug 2;16(8):e0255423. doi: 10.1371/journal.pone.0255423 (PMC8328323; doi:10.1371/journal.pone.0255423)
Supplement: S4 Fig — (PDF) [file pone.0255423.s006.pdf]

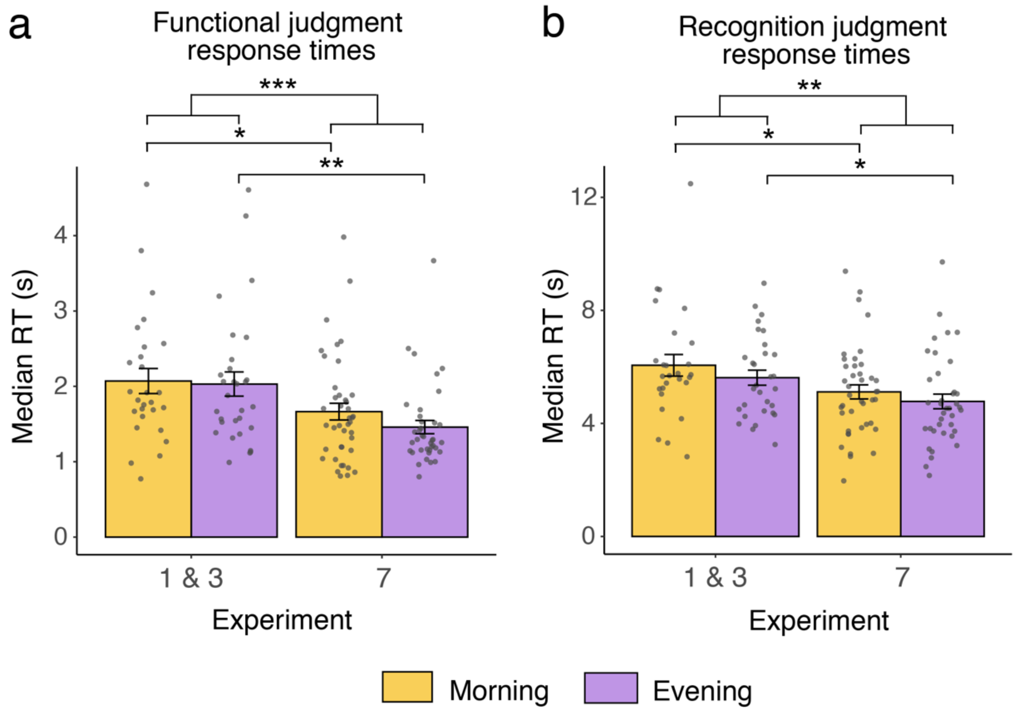

S4 Fig. Overall slider response times. Median slider response time differences in Experiments 1 and 3 (pooled; E1&3) versus Experiment 7 (E7) for (a) functional judgments and (b) recognition judgments. ***$p < .001$, **$p < .01$, *$p < .05$.
