# Supplementary material for: Examining the effects of time of day and sleep on generalization
Source: PLoS One. 2021 Aug 2;16(8):e0255423. doi: 10.1371/journal.pone.0255423 (PMC8328323; doi:10.1371/journal.pone.0255423)
Supplement: Attachment — Submitted filename: Response to Reviewers.pdf [file pone.0255423.s008.pdf]

Response to Reviewers

Thank you for submitting your manuscript to PLOS ONE. After careful consideration, we feel that it has merit but does not fully meet PLOS ONE's publication criteria as it currently stands. Therefore, we invite you to submit a revised version of the manuscript that addresses the points raised during the review process. In addition to the comments from the reviewers, I have additional suggestion for you to consider. Given that a significant portion of this research specifically focused on the impact of sleep on generalisation, it would be advantageous if the title was modified to include sleep and not just time-of-day.

We thank you and the Reviewers for your time and very valuable feedback. We have made substantial revisions to the manuscript and believe it is now a much stronger paper.

We agree that incorporating "sleep" into the manuscript title would be useful. We have modified the title to: "Examining the effects of time of day and sleep on generalization"

Reviewer #1: This is a very long, complex paper which uses a very complicated task to assess whether or not and to what degree people generalize from exemplars. It provides, across a series of studies, a reasonably clear message about sleep (it doesn't seem to influence generalization) and a somewhat less strong message about time of testing (there is greater generalization in the morning than in the evening). The authors do an excellent job of explaining their complex patterns of results and make clear the connections to existing literature (i.e., the references and the explanations) are excellent. The discussion is particularly strong and may well be influential for subsequent work.

what would have made this paper a better paper? If it were possible to focus/limit analyses to DVs relevant to generalization, it would have been a far easier read, especially given the large number of studies included. On the other hand, this is an extremely honest paper which allows readers to see warts in the research process.

We thank the Reviewer for their positive and helpful evaluation. We agree that the inclusion of several DVs, while providing a complete description of the data, may come at the cost of less clarity in the paper's main messages. To address this, we have moved a summary table (originally Supplementary Table 1) into the main text and added two new columns which summarize the time of day effect on generalization for each experiment. We also added a row at the bottom displaying the overall time of day effect in the experiments with matched generalization paradigms (Experiment 1,3 & 7 combined). We believe this will allow readers to more readily appreciate the primary generalization results across experiments.

Table 1. Experiment design and time of day effects on generalization.

	Experiment Design								Time of day effect on generalization	
	**Number of Sessions**	**Delay Between Sessions**	**Session 1 Start Time**	**Session 2 Start Time**	**Bridge Exposure**	**Exemplars Per Category**	**Number of Test Trials**	**Order of Tasks**	**Functional judgment *p***	**Recognition *p***
**Exp. 1**	2	12 hours	Morning: 9AM Evening: 8PM	Morning: 9:30PM Evening: 9AM	To criterion	5	53		.003**	.06§
**Exp. 2**	2	12 hours	Morning: 9AM Evening: 8PM	Morning: 9:30PM Evening: 9AM	Morning: 1 block Evening: 6 blocks	5	53		.44	.52
**Exp. 3**	2	24 hours	Morning: 9AM Evening: 8PM	Morning: 9AM Evening: 8PM	To criterion	5	53		.0003***	.01*
**Exp. 4**	1		4PM		To criterion	5	53			
**Exp. 5**	1		Morning: 9AM Evening: 8PM		To criterion	3	21	RAT, Distractor Priming, Generalization	.08§	.18
**Exp. 6**	1		Morning: 9AM Evening: 8PM		To criterion	3	21	Generalization, RAT, Distractor Priming	.47	.34
**Exp. 7**	1		Morning: 9AM Evening: 8PM		To criterion	5	53	Generalization, RAT, Distractor Priming	.56	.93
**Exp. 1, 3, & 7 Combined**			Morning: 9AM Evening: 8PM		To criterion	5	53		.0007***	.04*

Number of Sessions = number of experimental sessions; two session experiments had a second test administered after a delay. Delay Between Sessions = approximate time between Session 1 and Session 2. Session 1 Start Time = approximate start time of Session 1 for each group. Session 2 Start Time = approximate start time of Session 2 (referred to in text as Test 2) for each group. Bridge Exposure = number of blocks (10 trials per block) that participants encountered bridge satellites during the training phase. Criterion was reached when a participant achieved nine out of ten trials in a block correct. In Experiment 2 the number of blocks was different between groups, reflecting our aim to match initial generalization performance in this experiment. Exemplars Per Category = the number of unique satellites in each of the three categories encountered during the training phase. Number of Test Trials = total number of trials during the test phase in one session. Order of Tasks = the order of the tasks that participants completed in experiments that administered additional measures (RAT, Distractor Priming). Generalization refers to the full generalization paradigm (i.e. both training and test). Functional judgment p = Difference between morning and evening group for functional judgment generalization. Recognition p = Difference between morning and evening group for recognition generalization. ***$p < .001$, **$p < .01$, *$p < .05$, §$p < .1$

Reviewer #2: This study investigates a very interesting phenomena, (specifically, generalization of newly acquired memories) that sleep would appear to be a likely candidate to facilitate/support/enhance. The inclusion of a nap control experiment provides valuable insight, and is something that is not normally part of experimental designs that include sleep, so this welcome addition allows for valuable comparisons between a night of sleep and a nap. Along the same line, additional behavioural experiments to further probe time of day add rigor to the overall report, despite that some effects were not easily replicated, highlighting the inherent complicated nature of studying this phenomena and the pitfalls of overlooking time-of-day effects.

We greatly appreciate the Reviewer's comments and helpful suggestions. To address their concerns, we have modified language throughout the paper and report new analyses. We believe the paper has significantly improved as a result of these changes.

Major comments:

1. One of the main conclusions is difficult to support based on the approach and outcomes, specifically, statements such as: "…suggesting a role for circadian rhythms apart from sleep". It cannot be determined if circadian rhythms, per se, or other factors linked to time of day, or time elapsed between test and retest explain the effect of time on generalization. This conclusion is also not supported by / consistent with the analyses on chronotype. Suggest removing this or modifying these statements to be more in line with the study design and results.

We completely agree with the Reviewer that this study does not directly support a role for circadian rhythms and that we should be more careful with the language on this point. We have modified several statements throughout the paper to address this issue. Namely, any mention of "circadian rhythms" as an explanation of our results was replaced with the broader term "time of day". The following statements that initially mentioned circadian rhythms as an explanation now read as follows:

"In a nap experiment, we found that generalization did not benefit from having slept recently, suggesting a role for **time of day** apart from sleep."

"A nap study suggested that this effect is likely due to the particular **time of day**, as opposed to having slept recently (Experiment 4)."

"To more directly disentangle a restorative sleep account from a **time of day** account, we ran a nap study"

"Thus, the morning-time benefit for generalization cannot be attributed to the Morning groups having slept recently, implicating a more general role of **time of day**."

"Taken together, these experiments suggest that the **time of day** influences this form of generalization."

2. Another main conclusion: "…a state of lowered inhibition in the morning may facilitate spreading activation between otherwise separate memories…" is difficult to substantiate, as "inhibition" was not directly measured in the current study. Suggest framing this more as a possibility to be investigated in the future, rather than an interpretation that can be made from the current study.

We agree that the statements about inhibition need to be qualified given that we did not directly measure inhibition. We edited this particular statement mentioned by the Reviewer, as well as several other statements about inhibition throughout the paper, to be more speculatory in nature (bolded reflect changes in wording):

"We **suggest** that a state of lowered inhibition in the morning may facilitate spreading activation between otherwise separate memories, promoting this form of generalization."

"Evidence that off-peak circadian states **may be** associated with low inhibition include..."

"Consistent with theories arguing that reduced cognitive control can be adaptive [65,66], **we suggest that** generalization may be another instance where low inhibition is an asset [67]"

"Moderate instead of strong activation of related satellites during the study of the bridge items, **possibly** due to higher inhibition in the evening than morning, could result in repulsion instead of integration of the bridged categories."

We also added the following sentence to the General Discussion:

"Although we have suggested that changes in inhibition may underlie the time of day effect on generalization, we did not assess neural inhibition directly in this study. An important avenue for future research will be to **more directly e**stablish how time of day changes in neural excitation and inhibition relate to behavioral outcomes. For instance, our research, combined with other behavioral evidence **suggesting** reduced inhibition in the morning,"

3. Null results are difficult to interpret; for which there are many across the series of Experiments. It would be helpful to get a sense of whether the null findings were due to insufficient statistical power, re; ~ 15/group is common in the literature, but often questionable whether sufficient to detect variable/subtle behavioural effects. If power analyses were conducted, they should be presented, otherwise, it would be helpful to know if the study was adequately powered. The pooled analysis presented in Experiment #7 partly speaks to this issue, but not expressly for each individual experiment.

We agree with the Reviewer that establishing whether or not statistical power was met will help in interpretation of the null results. To examine whether the null time of day effects were due to lack of statistical power, we conducted a power analysis based on the time of day effect on functional judgment generalization observed in Experiment 1. We used these data because time of day effects on generalization have not been

studied in prior literature. The time of day effect on generalization in Experiment 1 had a large effect size: Cohen's *d* = 1.20. The power analysis indicated that a sample of 13 participants per group would provide over 90% power to detect differences in group means at an alpha of .05. Given that the sample sizes in all of our subsequent experiments met or exceeded this number, it is unlikely that the lack of time of day effect observed in some of the subsequent experiments were due to insufficient statistical power. To make this transparent to readers we added the following statement in the Methods section for Experiment 2:

"Given the effect size for functional judgment generalization observed in Experiment 1 (Cohen's *d* = 1.20), this sample provides over 90% power to detect the same effect."

4. In Experiment #1, several correlations between outcome variables were performed, however, it is not clear how many such analyses were performed, and if this was done in any systematic/parsimonious manner and seem overly exploratory the way they are presented. Multiple comparisons may be an issue if many analyses were conducted without any correction. This also applies to subsequent experiments.

We compiled a list of all the correlations conducted between outcome variables for each experiment:

<u>Experiment 1:</u>

- Functional judgment generalization x Recognition generalization

<u>Experiment 5, Experiment 6</u>

- Functional judgment generalization x RAT
- Recognition generalization x RAT Functional judgment generalization x Distractor priming
- Recognition generalization x Distractor priming
- RAT x Distractor priming

<u>Experiment 7</u>

- Functional judgment generalization x RAT
- Recognition generalization x RAT
- Functional judgment generalization x Distractor priming
- Recognition generalization x Distractor priming
- RAT x Distractor priming
- Functional judgment generalization x Trained functional judgments*

These were our *a priori* correlations of interest, except for the one correlation in Experiment 7 that is indicated with an asterisk (*). All except two of the correlations we ran assessed relationships between performance on generalization, the RAT, and the distractor priming task. Our decision to administer the RAT and distractor priming task was to specifically assess how performance on these tasks, which have previously shown increased performance in the morning, relate to the morning-time benefit we

observed for generalization. We agree that the way these correlations were presented did not make it clear that these correlations were hypothesized and performed systematically. To address this, the following paragraph from Results section on Experiment 5 (the first experiment where we ran these additional measures) was modified (changes in bold):

"How might generalization relate to other tasks known to be better at non-optimal times of day, such as insight problem-solving on the Remote Associates Test (RAT; [63,64] and memory for distractors [37,50]? In this experiment, participants completed these tasks prior to the generalization paradigm. In order to keep the overall experiment length similar with the addition of these two tasks, we also used a simplified generalization paradigm with three instead of five exemplars per category, as well as fewer test trials. **We hypothesized that there would be positive across-subject correlations between generalization and these additional tasks.**"

The one exploratory correlation that we performed was in Experiment 7 between Functional judgment generalization and Trained functional judgments. We believe the exploratory nature of this correlation should be transparent to readers with this text from the Experiment 7 Results section:

"Post-hoc correlations revealed that trained functional judgments positively correlated with generalization for the Morning group ($r = 0.44$, $p = .004$), but there was no significant relationship for the Evening group ($r = -0.22$, $p = .18$). Though these analyses are exploratory, they suggest..."

5. There are a large number of experiments presented, with similar, but not exactly the same analysis strategies employed, with a very large number of null results presented alongside significant findings. It is easy for the main message to get buried in all this. Summary tables of results might help with the presentation.

We thank the Reviewer for this very useful suggestion. We have modified Supplementary Table 1, which was originally a summary table of design differences, to now include new two result columns which describe the main results for each study. We also added an additional row showing the overall time of day effect in the experiments with matched generalization paradigms (Experiment 1,3 & 7 combined). To make this more accessible to readers, we have moved the table into the main text (Table 1). We anticipate this will make the main message of the paper more clear.

Table 1. Experiment design and time of day effects on generalization.

	Experiment Design								Time of day effect on generalization	
	**Number of Sessions**	**Delay Between Sessions**	**Session 1 Start Time**	**Session 2 Start Time**	**Bridge Exposure**	**Exemplars Per Category**	**Number of Test Trials**	**Order of Tasks**	**Functional judgment *p***	**Recognition *p***
**Exp. 1**	2	12 hours	Morning: 9AM Evening: 8PM	Morning: 9:30PM Evening: 9AM	To criterion	5	53		.003**	.06§
**Exp. 2**	2	12 hours	Morning: 9AM Evening: 8PM	Morning: 9:30PM Evening: 9AM	Morning: 1 block Evening: 6 blocks	5	53		.44	.52
**Exp. 3**	2	24 hours	Morning: 9AM Evening: 8PM	Morning: 9AM Evening: 8PM	To criterion	5	53		.0003***	.01*
**Exp. 4**	1		4PM		To criterion	5	53			
**Exp. 5**	1		Morning: 9AM Evening: 8PM		To criterion	3	21	RAT, Distractor Priming, Generalization	.08§	.18
**Exp. 6**	1		Morning: 9AM Evening: 8PM		To criterion	3	21	Generalization, RAT, Distractor Priming	.47	.34
**Exp. 7**	1		Morning: 9AM Evening: 8PM		To criterion	5	53	Generalization, RAT, Distractor Priming	.56	.93
**Exp. 1, 3, & 7 Combined**			Morning: 9AM Evening: 8PM		To criterion	5	53		.0007***	.04*

Number of Sessions = number of experimental sessions; two session experiments had a second test administered after a delay. Delay Between Sessions = approximate time between Session 1 and Session 2. Session 1 Start Time = approximate start time of Session 1 for each group. Session 2 Start Time = approximate start time of Session 2 (referred to in text as Test 2) for each group. Bridge Exposure = number of blocks (10 trials per block) that participants encountered bridge satellites during the training phase. Criterion was reached when a participant achieved nine out of ten trials in a block correct. In Experiment 2 the number of blocks was different between groups, reflecting our aim to match initial generalization performance in this experiment. Exemplars Per Category = the number of unique satellites in each of the three categories encountered during the training phase. Number of Test Trials = total number of trials during the test phase in one session. Order of Tasks = the order of the tasks that participants completed in experiments that administered additional measures (RAT, Distractor Priming). Generalization refers to the full generalization paradigm (i.e. both training and test). Functional judgment p = Difference between morning and evening group for functional judgment generalization. Recognition p = Difference between morning and evening group for recognition generalization. ***$p < .001$, **$p < .01$, *$p < .05$, §$p < .1$

6. The conclusion (albeit stated as exploratory), that the “lack of replication of the time of day effects in Experiment 7 may be due to this cohort of participants taking the task less seriously” is unfounded. How was the “seriousness” of the participants ascertained?

We agree this statement was unclear. We have now altered this sentence to operationalize our use of the term “seriousness”:

“Though exploratory, these analyses suggest that the lack of replication of the time of day effects in Experiment 7 may be due to this cohort of participants taking the task less seriously**: they had faster and less variable responses, in addition to weaker functional judgments for trained satellites (which were defined for the participants to be functional, suggesting poor instruction-following)**.”

Minor comments:

1. While the initial aim of the first 3 experiments was to investigate sleep, and the hypotheses are presented only very briefly in the introduction. The subsequent studies were designed to investigate why no effect of sleep was observed. The Introduction could benefit from presenting specific hypotheses for the subsequent studies.

We believe that reworking the introduction to include all the hypotheses is challenging for the paper’s structure, especially given the many design changes and number of studies. We hope that flagging sleep as important in our new title (“Examining the effects of time of day and sleep on generalization”), and the interim introductions throughout the results section will work to guide readers through our hypotheses and motivations for each of the subsequent studies.

2. How many participants reached the 1-hour cut-off for the training phase? Performance in the case where they were cut-off due to the time limit may be interpreted differently than those who completed the training in the allotted time.

We thank the Reviewer for bringing up this point. We agree that this information is important and should be reported in the paper. We reexamined the data to determine how many participants reached the 1-hour training cutoff. Across all experiments, 10.27% of participants did not reach criterion after 1-hour of training. The proportion of participants in each experiment who did not reach criterion by the end of training are as follows:

Experiment 1: 10.00% (3 participants)
Experiment 2: 11.11% (3 participants)
Experiment 3: 3.70% (1 participant)
Experiment 4: 13.51% (5 participants)
Experiment 5: 4.65% (2 participants)
Experiment 6: 4.08% (2 participants)
Experiment 7: 17.72% (14 participants)

To make this available to readers, we added a column reporting the number of participants in each study who did not meet criterion after 1-hour of training in Supplementary Table 1 (originally Supplementary Table 2).

S1 Table. Training performance.

		Number of blocks to criterion		Feature accuracy last block		Class accuracy last block	Participants who did not meet training criterion
		**Initial**	**Bridge**	**Initial**	**Bridge**	**Initial**	**Initial**
**Exp. 1**	Morning	4.15±0.36	2.46±0.27	0.73±0.02	0.95 ±0.01	0.94±0.03	1
	Evening	4.94±0.50	2.59±0.32	0.73±0.02	0.94±0.01	0.96±0.01	2
	Group Diff. *p*	.24	.77	.96	.56	.63	
**Exp. 2**	Morning	4.36±0.48	1 (fixed)	0.71±0.02	0.69±0.06	0.98±0.01	1
	Evening	3.77±0.46	6 (fixed)	0.73±0.03	0.98 ±0.01	0.98±0.01	2
	Group Diff. *p*	.38		.59	.000005***	.61	
**Exp. 3**	Morning	4.07±0.32	2.71±0.27	0.73±0.02	0.94±0.01	0.96±0.01	1
	Evening	4.38±0.27	2.54±0.31	0.76±0.01	0.96±0.01	0.97±0.01	0
	Group Diff. *p*	.46	.67	.38	.35	.56	
**Exp. 4**	Nap	4.45±0.44	2.10±0.14	0.76±0.02	0.97±0.01	0.98±0.01	2
	No Nap	3.94±0.39	2.52±0.23	0.68±0.04	0.95±0.01	0.96±0.02	3
	Group Diff. *p*	.40	.11	.07	.30	.65	
**Exp. 5**	Morning	4.36±0.41	2.28±0.11	0.77±0.02	0.95±0.01	0.98±0.01	2
	Evening	4.39±0.45	1.94±0.17	0.76±0.02	0.97±0.01	0.98±0.01	0
	Group Diff. *p*	.96	.09	.72	.35	.75	
**Exp. 6**	Morning	4.12±0.33	2.31±0.16	0.74±0.01	0.96 ± 0.01	0.97±0.01	1
	Evening	5.57±0.47	2.52±0.23	0.76±0.02	0.95 ± 0.01	0.96±0.02	1
	Group Diff. *p*	.01*	.43	.17	.50	.77	
**Exp. 7**	Morning	4.34±0.22	2.17±0.11	0.68±0.02	0.95±0.01	0.96±0.01	9
	Evening	3.74±0.21	2.21±0.11	0.70±0.02	0.97±0.01	0.94±0.02	5
	Group Diff. *p*	.05*	.80	.67	.07	.31	

Measures of performance during the main training phase (Initial) and the following bridge training phase (Bridge) are shown for each experimental group. Number of blocks to criterion = The number of blocks it took to reach the training criterion (Initial training criterion was 66% for feature accuracy and 90% class accuracy), or the number of blocks after 1-hour had passed; Bridge training criterion was 90% feature accuracy. Feature accuracy last block = Proportion of features correct in the last block of the initial and

bridge training phase. Class accuracy last block = Proportion of trials correct on class naming in the last block of the initial training phase. Participants who did not meet training criterion = The number of participants that did not reach the training criteria in the initial training phase after 1-hour had passed. Descriptive statistics (mean ± one SEM) and group differences (p-values from t-test) are shown. Note that the number of trials per block in the initial training phase was lower in Exp. 5 and Exp. 6. ***$p < .001$, *$p < .05$.

For the Reviewer's information, we ran the t-tests between morning and evening groups on both of our generalization measures for each experiment with these subjects excluded, and found no substantive impact on the results.

3. Partial credit on the RAT was given using Levenshtein distance, which is innovative, but perhaps not appropriate, given that the goal of the task is not to assess spelling/typographical errors. i.e., this measure confounds semantic accuracy with typographical precision (which is not of interest in this case). It might be more appropriate to instead manually assess whether errors were typographical, in which case, effectively the correct word (full points), or an incorrect word (no points). If a large number of responses were partial scores, and if in most cases the Levenshtein distance is neither close to a full point nor zero, then this may be problematic. By contrast, this may not be an issue for the priming score in the distractor priming task, re; lexical accuracy is relevant in that case.

We agree with the Reviewer that scoring the RAT should ideally not penalize for spelling/typographical errors. To address the Reviewer's concern about a large number of RAT responses being partial scores, we calculated the percentage of RAT responses that were assigned a partial credit via Levenshtein distance. This revealed that only 2.5% of the RAT responses (averaged across all three experiments) were assigned partial scores. Although this meant only a very small proportion of answers were assigned partial credit, we wanted to confirm that this did not have an impact on our results. To address this, we had a coder (blind to condition) manually rate partial score responses as either correct (full points) or incorrect (no points). We then reran the statistical tests examining time of day effects on the RAT using these manual scores and found that it did not change the results. We added the following statement in the methods section:

"Only 2.5% of participant responses were assigned partial credit using this method. Manually scoring these partial credits as either correct or incorrect for semantic accuracy (ignoring spelling and typographical errors) had no material impact on the results."

4. The results of the earlier experiments were not replicated, which was attributed to the changes in the task design. This is intriguing as it would suggest some sort of dose-response relationship for the shorter version. Suggest mentioning this possibility in the Discussion.

The Reviewer raises an interesting point. While we did discuss that the time of day effect may be sensitive to category structure (namely whether there were 3 exemplars or 5 exemplars per category), we added the following conclusion sentence to make this

point about task design more clear. This sentence was added to the end of the first paragraph of the General Discussion section titled “Time of day effects may be sensitive to category structure and task engagement”:

“We did not find a time of day effect on generalization in a simplified version of our task that involved fewer exemplars per category. Consistent with participants having superior exemplar memory in this version, it is possible that with fewer exemplars participants were simply memorizing the features of each satellite with less sensitivity to the shared structure of the categories, thereby weakening generalization. In support of this possibility, several category learning studies have demonstrated that exemplar variability is particularly important for generalization [68,81–84]. Indeed, neither Morning nor Evening groups exhibited reliable generalization in the experiments with fewer exemplars. **In sum, the finding suggests that the strength of time of day effects on generalization may depend on features of the task design and task complexity.** Another possibility is that the simplified paradigm lacked adequate sensitivity to detect generalization during the test phase, which had less variable items and substantially fewer total trials.”

5. The Discussion is very long, particularly given that there are already “interim” discussions for each experiment, and also in light of the large volume of null (and somewhat inconclusive) results. Suggest significantly shortening this section to summarize the main message.

We agree with the Reviewer that the discussion is lengthy. However, we believe that given the number of experiments and inconsistent results to consider, its length is warranted. Reviewer 1 pointed to the discussion as a strength of the paper in its potential to inspire future research, which we believe is especially important given our complex findings that may be relevant to researchers in several different fields.
